# Anandamide Exerts a Differential Effect on Human Placenta Before and After the Onset of Labor

**DOI:** 10.3389/fphys.2021.667367

**Published:** 2021-05-21

**Authors:** Paula Accialini, Cyntia Abán, Tomás Etcheverry, Mercedes Negri Malbrán, Gustavo Leguizamón, Vanesa Herlax, Sabina Maté, Mariana Farina

**Affiliations:** ^1^Laboratorio de Fisiopatología Placentaria, Facultad de Ciencias Médicas, Centro de Estudios Farmacológicos y Botánicos (CEFyBO – CONICET – UBA), Universidad de Buenos Aires, Ciudad de Buenos Aires, Argentina; ^2^Centro de Educación Médica e Investigaciones Clínicas Norberto Quirno (CEMIC), Ciudad de Buenos Aires, Argentina; ^3^Laboratorio de Estudios de la Interacción Toxinas Proteicas - Membranas, Instituto de Investigaciones Bioquímicas de La Plata (INIBIOLP - CONICET), Facultad de Ciencias Médicas, Universidad Nacional de La Plata, La Plata, Argentina

**Keywords:** anandamide, prostaglandins, nitric oxide synthase, placenta, labor

## Abstract

The onset of labor involves the action of multiple factors and recent reports have postulated the endocannabinoid system as a new regulator of this process. Our objective was to study the role of anandamide, one of the main endocannabinoids, on the regulation of placental molecules that contribute to the onset of labor at term. Placental samples were obtained from patients with laboring vaginal deliveries and from non-laboring elective cesarean sections. Vaginal delivery placentas produced higher prostaglandins levels than cesarean section samples. Besides, no differences were observed in NOS basal activity between groups. Incubation of vaginal delivery placentas with anandamide increased prostaglandins concentration and decreased NOS activity. Antagonism of type-1cannabinoid receptor (CB1) did not alter the effect observed on NOS activity. Conversely, incubation of cesarean section placentas with anandamide reduced prostaglandins levels and enhanced NOS activity, the latter involving the participation of CB1. Furthermore, we observed a differential expression of the main components of the endocannabinoid system between placental samples, being the change in CB1 localization the most relevant finding. Our results suggest that anandamide acts as a modulator of the signals that regulate labor, exerting differential actions depending on CB1 localization in laboring or non-laboring term placentas.

## Introduction

The onset of term human labor involves the action of a variety of factors that enables the transition from uterine quiescence to contractility. Indeed, an accurate interplay among different mediators produced by the placenta, mother, and fetus is necessary to trigger the initiation and progression of labor. In the last years extensive research has attempted to elucidate the factors involved in the mechanism of parturition. In line with this, several reports have postulated the endocannabinoid system (ES) as a new member of the complex range of factors that regulate the onset of labor. The ES is a signaling system that comprises the endogenous bioactive lipids called endocannabinoids (ECs); the cannabinoid receptors (CBs) and the enzymes involved in their metabolism. It was demonstrated that, in human gestation at term, plasma concentration of anandamide (N-arachidonoylethanolamine, AEA), one of the most extensively studied endocannabinoids, is higher in laboring than in non-laboring women (Habayeb et al., 2004). Furthermore, the human placenta produces higher levels of AEA compared to other reproductive tissues (Marczylo et al., 2010). After its synthesis, AEA can act as an endogenous ligand of the CBs, which are inhibitory G protein coupled receptors (GPCRs) (Howlett, 2005). Particularly, type-1 CB (CB1) has been localized in human term placentas (Park et al., 2003). Appropriate desensitization of CB1 is a critical process in its regulation and it may occur by endocytosis (Rozenfeld, 2011; Wickert et al., 2018). Lipid raft and caveolae, which are plasma membrane subdomains, have been described as regulators of GPCRs signaling (Moffett et al., 2000). Particularly caveolin-1, which is the most relevant structural protein of caveolae (Patel and Insel, 2009), can act as a regulator of CB1 by internalizing this receptor via caveolae-related endocytosis (Lefkowitz, 1998).

During the third trimester, the contractile activity of the uterus gradually increases. Thus an increment in the factors associated with myometrial contraction is expected. It is well-known that prostaglandins (PGs) play a pivotal role in the process of labor since they stimulate myometrium contractility; cervical ripening and membrane rupture (Challis et al., 1997, 2002, 2009). Several factors are known modulators of placental PGs production (Carbillon et al., 2000; Keelan et al., 2000; Challis et al., 2001), and in the last years mounting evidence suggest that the ECs are also involved in its regulation (Mitchell et al., 2016).

Initiation of labor also involves profound hemodynamic changes. The placenta capability to produce vasoactive mediators and therefore to regulate placental circulation is cornerstone in this process (Myatt, 1992). The uterine contractions that occur during labor are associated with intermittent utero-placental perfusion, suggesting that changes in the level of factors controlling blood flow would contribute to the triggering of labor (Cindrova-Davies et al., 2007). Among these factors, nitric oxide (NO) is a well know auto/paracrine vasodilator agent that contributes to the reduction of the vascular resistance and the modulation of the uteroplacental and fetal circulation (Sladek et al., 1997). Nitric oxide production and NOS activity are strongly regulated by multiple factors. Particularly, AEA can stimulate or inhibit NOS activity and/or NO production through CBs activation depending on tissue context and cell type (Cella et al., 2008; Sordelli et al., 2011, 2012; Aban et al., 2013).

Even though the relationship between AEA action and the synthesis of PGs and NO has been previously demonstrated, its precise role as a regulatory molecule of the placental signals related with the onset of human labor has not been clarified.

For the aforementioned, in the present work we aimed to elucidate the participation of the ES in the regulation of PGs production and placental NOS activity in human placenta at term.

## Materials and Methods

### Placental Samples and Tissue Collection

This study was approved by the Ethics Committee of the Center for Medical Education and Clinical Research “Norberto Quirno” (N° 684) and all patients signed informed consent.

Placental tissue was obtained from healthy normotensive women with uncomplicated pregnancies at term (37–40 completed weeks of gestation). Samples were divided into two groups: *n* = 28 were vaginal deliveries (VD) and progressed satisfactorily to a non-instrumented birth; *n* = 27 were non-laboring elective cesarean sections (CS). The characteristics of the patients are summarized in Table 1. Onset of labor was defined as the initiation of regular uterine activity, leading to cervical dilation and effacement. Women treated with oxytocin for induction of labor and women with intrapartum cesarean section were excluded from the study. All placental samples were collected and processed within 1 h after delivery. Following removal of blood vessels or clots, tissue was randomly cut into small pieces, repeatedly washed with saline solution to remove the excess blood, and finally either processed or stored at −80°C.

**TABLE 1:** Clinical characteristics of the patients.

Parameter	Cesarean section (*n* = 27)	Vaginal delivery (*n* = 28)
Mean gestational age (weeks)	38.6 ± 0.2	40.0 ± 0.8
Mean maternal age (years)	34.8 ± 1.75	35.7 ± 1.9

*Values are expressed as the mean ± SEM. Patients from both groups have no previous history of hypertension, gestational diabetes, autoimmune diseases, multifetal gestation, or fetal anomalies.*

### Prostaglandins Concentration Measurement

Prostaglandin E2 (PGE2) and prostaglandin F2a (PGF2a) were measured in placental explants from VD or CS as described previously (Ribeiro et al., 2004; Cella et al., 2010). Briefly, tissues were incubated for 60 min in Krebs-Ringer bicarbonate buffer (5% v/v Krebs solution; 2.0 g/L glucose; 2.1 g/L NaHCO_3_) at 37°C in a 5% CO_2_ atmosphere. After incubation, placental tissues were used for total protein content measurement by Bradford technique. Supernatants were acidified and then PGs were extracted twice with ethyl acetate. Prostaglandins concentration was determined by radioimmunoassay. Sensitivity was 5–10 pg/ml, and values are expressed as pg PGs/mg of protein.

#### Incubation With AEA and Met-AEA

Fresh explants from VD or CS placentas were cultured with 10^–5^ mol/L, 10^–7^ mol/L, or 10^–9^ mol/L AEA (Biomol, Miami, United States) y el), or R-(+)-Methanandamide (Met-AEA, Sigma, St. Louis, United States), a stable AEA analogous, for 24 h in RPMI 1640 culture medium (Microvet, Buenos Aires, Argentina) supplemented with 10% v/v bovine fetal serum (Natocor, Córdoba, Argentina) and gentamicin (50 μg/ml). Additionally, as a positive control of PG synthesis, placental explants were incubated with LPS (lipopolysaccharide from *E. coli*, 1 mg/ml). After 24 h of culture, PGs concentration was determined as abovementioned.

### NOS Activity Measurement

NOS activity was determined by the modified technique of Bredt and Snyder (Aban et al., 2013), which measures the conversion of [^14^C]-arginine into [^14^C]-citrulline. Tissues were homogenized in Hepes buffer (0.45 mmol/L CaCl_2_; 25 mmol/L valine and 100 mmol/L DTT) and then 0.12 mmol/L NADPH and 10 μmol/L [^14^C]-arginine (0.3 μCi) (Cayman Chemical, Ann Arbor, United States) were added to the homogenate. Samples were incubated at 37°C in a 5% CO_2_ incubator for 15 min and then centrifuged at 7,800 g for 15 min at 4°C. The supernatant was eluted in a DOWEX 50WX8 column ion-exchange resin (Na^+^ form) (Biorad, Buenos Aires, Argentina). The column was washed three times with PBS 1X (137.0 mmol/L NaCl; 2.7 mmol/L KCl; 8.1 mmol/L Na2HPO4; 1.47 mmol/L KH2PO4; pH = 7.4) and the radioactivity of the eluates was quantified by liquid scintillation counting (Beckman). NOS activity was expressed as fmol of [^14^C]-citrulline/mg of protein during 15 min.

#### Incubation With AEA

Fresh explants from VD or CS placentas were pre-incubated with 10^–7^ mol/L, 10^–8^ mol/L, or 10^–9^ mol/L AEA in Krebs buffer in a Dubnoff shaking incubator with a 5% CO_2_ atmosphere at 37°C for 30 min. After culture, explants were homogenized in Hepes buffer as abovementioned.

#### Incubation With AM 251

Fresh explants from VD or CS placentas were pre-incubated with 10^–7^ mol/L AM251 (CB1 antagonist, Tocris Cookson Inc., Ellisville, United States) in Krebs buffer in a Dubnoff shaking incubator with a 5% CO_2_ atmosphere at 37°C for 15 min. Then, 10^–7^ mol/L AEA was added and tissue was incubated 30 more minutes. After culture, explants were homogenized in Hepes buffer as mentioned before.

### Western Blot

Samples were prepared as previously described (Aban et al., 2013, 2016) and protein concentration was measured by Bradford assay. Lysates of placental explants (100 μg of protein) were boiled for 5 min and then electrophoresed through a SDS–polyacrylamide gel. The resolved proteins were transferred onto a nitrocellulose membrane. After incubating the blot in blocking solution [5% w/v non-fat dry milk in 1% v/v Tween-PBS (T-PBS)], membranes were rinsed with T-PBS and incubated overnight at 4°C with the appropriate primary antibody: FAAH 1:150 (gift from Dr. Benjamin Cravatt), N-acyl phosphatidylethanolamine phospholipase D (NAPE-PLD) 1:1,000 (Cayman Chemical Co., Ellsworth Road, MI, United States), CB1 1:250 (Biomol, Miami, United States), FLOTILIN 1:1,000 (Santa Cruz Biotechnology, California, United States), CAVEOLIN-1 1:500 (Santa Cruz Biotechnology), β-ACTIN 1:1,000 (Sigma, St. Louis, United States). Then the blots were rinsed with T-PBS and incubated with peroxidase-conjugate anti-Rabbit 1:10,000 (Jackson ImmunoResearch Laboratories, Sero-Immuno Diagnostics, INC, Tucker, GA, United States) at room temperature (RT) for 1 h. β-ACTIN was used as loading control. Proteins were visualized after incubation with enhanced chemiluminescence reagent (ECL, Sigma, St. Louis, United States) and light emission was detected by exposing the blots in an Image Quant 350 GE Healthcare. Protein content was quantified by densitometric analysis using ImageJ (NIH) program.

### Fatty Acid Amide Hydrolase (FAAH) Activity

FAAH activity was determined as established by Paria et al. (1996) with minor modifications. Placental tissue was homogenized and protein quantification was performed by Bradford assay. Then, 100 μg of protein were incubated for 15 min at 37°C in buffer 50 mmol/L Tris pH = 8.5 containing AEA and radio-labeled [^3^H]-AEA (0.05/μCi) (Perkin Elmer, Buenos Aires, Argentina). The reaction was stopped with a mixture of chloroform:methanol (1:1 v/v). After centrifugation, samples were resuspended in chloroform and spotted onto a thin chromatography Silica Gel 60 layer. The plate was exposed for 1 h in a saturated box with a mixture of acetic acid:ethyl acetate:hexane:distillated water (100:50:20:100 v/v). Standards of arachidonic acid (AA) and AEA were included as controls. Iodine was used to identify [^3^H]-AA, which is one of the products of FAAH activity. Radioactivity on the plate was counted in a scintillation counter by scrapping off the corresponding spots. Enzyme activity was reported as nmol [^3^H]-AA/mg protein/min.

### Immunohistochemistry

Placental tissue was fixed in 4% v/v paraformaldehyde overnight at 4°C. Then, samples were dehydrated by graded ethanol washes and embedded in paraffin. Slides were deparaffinized in xylene and rehydrated by graded ethanol. Tissue sections were permeabilized with 0.1% v/v Triton X-100 and endogenous peroxidase activity was blocked with 3% v/v H_2_O_2_. Sections were first incubated in blocking solution (2% non-fat dry milk in PBS) for 1 h at RT and then with CB1 (Biomol, Miami, United States) primary antibody diluted 1:50 in PBS, in a moist chamber overnight at 4°C. Then, slides were rinsed thrice in PBS and incubated with specific biotinylated anti-rabbit secondary antibody (1:200) for 1 h at RT. After washing the slides, sections were incubated with streptavidin-peroxidase complex and then stained with 0.05% v/v 3,3’-diaminobenzidine and counterstained with hematoxylin. Negative controls were obtained in the absence of primary antibody. Digital images were acquired using a camera (Nikon Corporation) mounted on a conventional light microscope (Nikon).

### RNA Isolation, Reverse Transcription, and Real-Time PCR

Total RNA was extracted using RNAzol reagent (Molecular Research Center, Cincinnati, United States) according to manufacturer’s instruction and RNA concentration was quantified using a NanoDrop (Thermo Fisher Scientific). One microgram of total RNA was converted into cDNA using M-MLV reverse transcriptase (Promega, Buenos Aires, Argentina) and random primers. The mRNA levels of *Faah, Nape-PLD*, and *Cb1* were quantitatively measured by qPCR on RG6000 (Corvette) using EasyTaq DNA polymerase (TransGen Biotech, Buenos Aires, Argentina) and EvaGreen dye (Biotium). The program used was: 5 min at 95°C (one cycle), followed by 40 cycles of 15 s at 95°C, 30 s at 60°C, and 1 min at 72°C for all primers. *Tata binding protein* (*Tbp*) was used as endogenous control, and negative control (no template) was included in all cases. The mRNA level of each gene was corrected to the level of *Tbp* and the relative expression was calculated using the 2^–ΔΔCt^ method and normalized to the ratio produced in the CS samples. Primers (Invitrogen, Buenos Aires, Argentina) sequences were: *Faah* (Fw: GCACACGCTGGTTCCCTTC, Rv: GGGTCCACGAAATCACCTTTGA); *Nape-PLD* (Fw: TCCC TCCAATAGATGCGGTCCT, Rv: TCCTCCCACCAGTCCAAC TCAA); *Cb1* (Fw: CCGATACACTTGGCATTGAC, Rv: GA CCGGGGTGTAAGAAGAAA); *Tbp* (Fw: CCCGAAACGCC GAATATAATCC, Rv: AATCAGTGCCGTGGTTCGTG).

### Apical (MVM) and Basal (BM) Membrane Vesicles Isolation

Human placenta villi from VD and CS term placentas were processed for MVM and BM enrichment as previously described (Levi et al., 2016). After obtaining the MVM and BM vesicles, detergent-resistant membranes (DRMs) were isolated by sucrose gradient centrifugation as performed previously (Levi et al., 2016). Sucrose gradient fractions of MVM and BM were dialyzed against TNE buffer (10 mmol/L Tris, 200 mmol/L NaCl, 1 mmol/L EDTA, pH 7.4) overnight in order to eliminate sucrose and finally subjected to SDS–PAGE polyacrylamide gels for protein detection by Western blot analysis.

### Statistical Analyses

Statistical analysis was performed using GraphPad Prism Software (San Diego, CA). Data are expressed as the mean ± SEM and were analyzed using Student’s *t*-tests or One-way ANOVA following Tukey’s multiple comparison tests as post-test. Values of *p* < 0.05 were considered significant.

## Results

### Analysis of PGs Concentration

**FIGURE 1:**
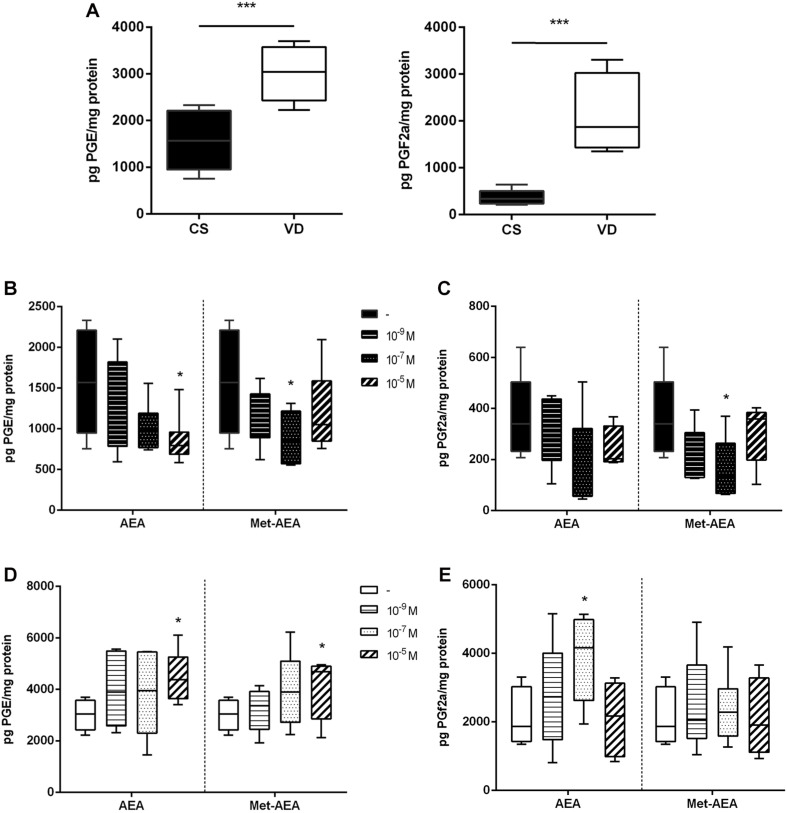
Analysis of prostaglandins concentration in term placenta. **(A)** Basal prostaglandin E (left panel) and F2a (right panel) production in cesarean section (CS) and vaginal delivery (VD) placentas (***P < 0.001, *n* = 10). **(B)** Prostaglandin E and **(C)** prostaglandin F2a production measured after culturing CS placentas with different concentrations of AEA or Met-AEA (*P < 0.05 vs. control, *n* = 10). **(D)** Prostaglandin E and **(E)** prostaglandin F2a production measured after culturing VD placentas with different concentrations of AEA or Met-AEA (*P < 0.05 vs. control, *n* = 10).

To elucidate if differences in the basal production of PGs exist between non-labor cesarean section (CS) and vaginal delivery (VD) placentas, PGE and PGF2a concentrations were measured. Vaginal delivery placentas produce higher PGE and PGF2a levels than CS samples (Figure 1A), suggesting that labor modulating molecules regulate placental PGs production.

Since AEA plasma levels increase in women with spontaneous (Habayeb et al., 2004) and induced (Nallendran et al., 2010) labor, and we postulate this molecule as a relevant participant in this process (Accialini et al., 2020), the effect of AEA on placental PGs synthesis was analyzed. For this purpose, CS and VD placentas were incubated with different concentrations of AEA and then PGE and PGF2a concentration was measured. It is important to point out that the molecular products resulting from AEA degradation can be enzymatically converted into PGs and prostamides (Peiris et al., 2017). These products are indistinguishable by radioimmunoassay, leading to difficulties in differentiating them. For this reason, to determine if our observations were a consequence of AEA incubation instead of AEA degradation, CS and VD placentas were also incubated with Met-AEA, a non-hydrolyzable AEA analogous. Incubation with 10^–5^ mol/L AEA decreased PGE concentration in non-labor CS placentas (Figure 1B), while PGF2a maintained its levels unchanged (Figure 1C). Conversely, incubation of labor VD placentas with 10^–5^ mol/L and 10^–7^ mol/L AEA increased PGE (Figure 1D) and PGF2a (Figure 1E) concentration, respectively. The effect of Met-AEA incubation on PGs production remained identical to that observed with AEA for both placental groups. As a positive control of PGs synthesis, placentas were incubated with LPS, which resulted in an increase in PGE and PGF2a concentration in both CS and VD placentas (data not shown).

### Characterization of NOS Activity

**FIGURE 2:**
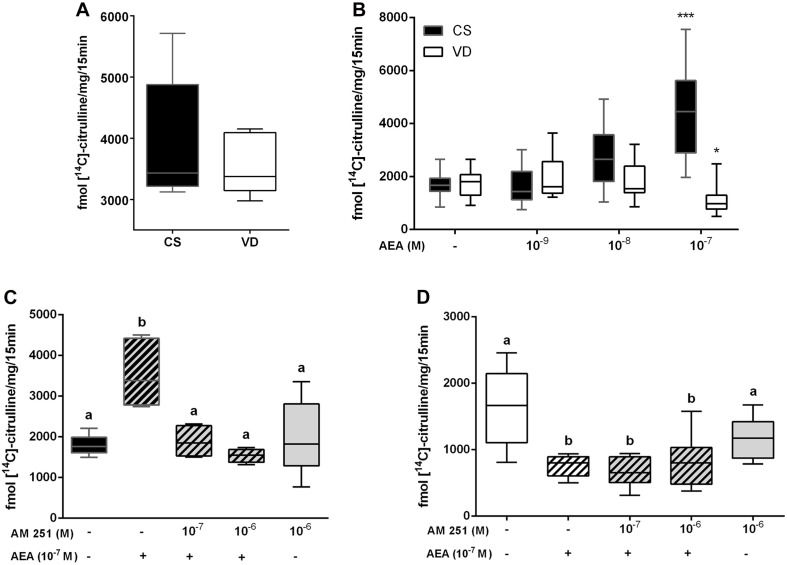
Characterization of nitric oxide synthase (NOS) activity in term placenta. **(A)** Basal NOS activity measured in CS and VD placentas (*n* = 8). **(B)** NOS activity measured after culturing CS and VD placentas with different concentrations of AEA (***P < 0.001, *P < 0.05 vs. control, *n* = 8). **(C)** NOS activity measured after culturing CS placentas and **(D)** VD placentas with 10^– 7^M AEA and different concentrations of AM 251. Different letters represent significant changes (*P* < 0.05 vs. control, *n* = 8) and the plain gray bar shows that AM 251 has no effect *per se*.

In line with our hypothesis that AEA acts as a key modulator in labor, previous results from our laboratory showed that AEA stimulates NOS activity through CB1 receptor in normal non-labor CS placentas (Aban et al., 2013). In the present work, NOS activity was analyzed comparing CS and VD placentas at term. Our results showed that there are no differences in the basal activity of this enzyme between groups (Figure 2A). However, incubation with 10^–7^ mol/L AEA produced a dual effect: while AEA enhanced NOS activity in CS samples, decreased this enzyme activity in VD placentas (Figure 2B).

In order to determine whether the dual effect that AEA has on NOS activity involves the participation of CB1, CS, and VD placental explants were co-incubated with AEA and AM251, a selective antagonist of CB1 receptor. Co-incubation with AEA + AM251 prevented the increment of NOS activity in CS samples (Figure 2C). Conversely, it does not alter the decrease observed in VD placentas (Figure 2D), suggesting that the inhibitory effect of AEA on NOS activity does not involve the participation of CB1.

### Expression of Relevant Members of the Endocannabinoid System in Human Placenta at Term

**FIGURE 3:**
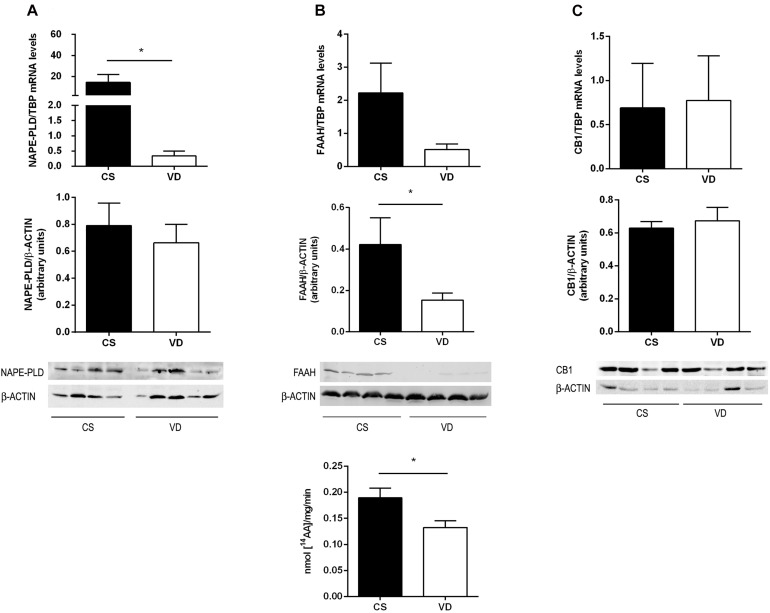
Expression of endocannabinoid system in term placenta. **(A)** NAPE-PLD mRNA levels and protein content, **(B)** FAAH mRNA levels, protein content and enzymatic activity, and **(C)** CB1 mRNA levels and protein content, measured in CS and VD placentas. Representative immunoblots of protein content are shown in the lower panel (*P < 0.05, *n* = 4–5).

The placenta expresses several components of the endocannabinoid system (ES) such as NAPE-PLD and FAAH (Aban et al., 2013), the main enzymes of AEA synthesis and degradation, respectively. Since AEA levels are modulated principally by these metabolic enzymes characterization of their expression was performed comparing CS and VD placentas at term. NAPE-PLD mRNA expression was lower in VD placentas compared to CS samples, while no differences were observed in its protein content (Figure 3A). Regarding FAAH, there were no differences in mRNA levels between groups, although a decrease in FAAH protein levels and enzymatic activity were observed in VD placentas (Figure 3B). This finding suggests that placental AEA concentration is higher in VD placentas. Lastly, CB1 receptor mRNA and protein levels were similar between groups (Figure 3C).

### Analysis of CB1 Localization

**FIGURE 4:**
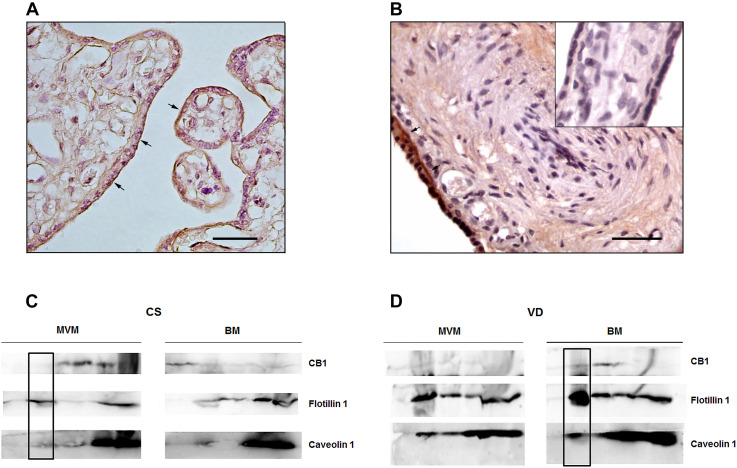
Analysis of CB1 localization in the syncytiotrophoblast of term placenta. Immunolocalization of CB1 in representative sections of placental villous from **(A)** CS placentas and **(B)** VD placentas. The arrows indicate specific CB1 staining and the inset shows the negative control. Magnification: 40×. Scale bar: 20 μm. **(C)** Differential distribution of CB1, Flotillin 1 and Caveolin 1 in sucrose gradient fraction of apical membrane (MVM) and basal membrane (BM) from CS placentas. CB1 is expressed in MVM in fractions different from fraction number 2. The inset shows that CB1 does not co-localize with Flotillin 1 and Caveolin 1 (*n* = 3). **(D)** Differential distribution of CB1, Flotillin 1 and Caveolin 1 in sucrose gradient fraction of MVM and BM from VD placentas. The inset shows that CB1 co-localizes with Flotillin 1 and Caveolin 1 in BM (*n* = 3).

Immunohistochemistry analysis revealed specific CB1 staining mainly in the apical membrane of the syncytiotrophoblast (STB) of CS placentas (Figure 4A). In VD samples, CB1 is localized in the STB cytoplasm (Figure 4B) although a slight staining is also observed in the basal membrane, suggesting that labor modulates CB1 localization. Accumulating evidence shows that CB1 can localize within LRs, which are known regulators of GPCRs signaling (Moffett et al., 2000). Particularly, LRs have a functional effect on CB1 as they regulate its positioning on the membrane and its intracellular trafficking (Sarnataro et al., 2005) via caveolae-related endocytosis (Lefkowitz, 1998). In order to study whether CB1 localizes within LRs in the STB, detergent-resistant membranes (DRMs) technique was performed in CS and VD placentas. Although DRMs are different from LRs (Herlax et al., 2009), they constitute a useful approach to investigate the interaction between membrane subdomains and proteins, as we have previously described (Levi et al., 2016).

The STB is a polarized cell formed by an apical membrane (MVM) that contacts the maternal circulation and a basal membrane (BM) that interacts with the fetal capillaries. For this reason, enrichment in MVM and BM vesicles from CS and VD placentas was performed and then DRMs were isolated from these samples. To improve protein detection, elutes were grouped in five fractions (see methodology), with fraction number two carrying the LRs. Protein expression of CB1, CAVEOLIN-1 and FLOTILIN-1, which are specific markers of caveolae and LRs, respectively, were analyzed in DRMs fractions of MVM and BM obtained from CS and VD placentas. Western blot analysis revealed that CB1 is expressed mainly in the MVM of CS placentas in fractions different from fraction number 2, so that CB1 is not co-localized with CAVEOLIN-1 and FLOTILIN-1 (Figure 4C). A slight CB1 band is also evidenced in BM samples (Figure 4C). However, in VD placentas CB1 is expressed in the BM and co-localizes with CAVEOLIN-1 and FLOTILIN-1 (Figure 4D).

These results, together with the observation by immunohistochemistry, suggest that labor promotes a differential location of CB1, within LRs.

## Discussion

The placenta is a dynamic organ that changes as pregnancy progresses and during the transition from uterine quiescence to contractility at the time of labor (Vannuccini et al., 2016). In the last years several reports have suggested a strong relationship between the endocannabinoid system (ES) and pregnancy performance, as its deregulation may lead to pathological conditions such as preeclampsia (Aban et al., 2013) and preterm labor (Wang et al., 2008; Bariani et al., 2015). In this work we demonstrate that changes in the expression and localization of members of the ES occur with the onset of labor, and that anandamide (AEA) differentially modulates prostaglandins (PGs) synthesis and nitric oxide synthase (NOS) activity in the human placenta at term.

During the third trimester, there is a progressive increase in the contractile activity of the uterus and therefore an increase in factors associated with muscle contraction such as PGs, is expected. Our results showed that vaginal delivery (VD) placentas have higher PGE and PGF2a concentration than non-labor cesarean section (CS) placentas.

Anandamide has been postulated as a relevant participant in labor (Habayeb et al., 2004; Nallendran et al., 2010). Previous results from our laboratory showed that this endocannabinoid contributes to the modulation of the oxytocin system in human placenta at term (Accialini et al., 2020). In line with this hypothesis, we observed a dual effect of AEA on PGs production, which suggests that the action of AEA depends on the placenta’s molecular context (labor vs. non-labor). Accordingly, Bariani et al. reported that AEA increases uterine PGF2a levels in a mouse model of preterm labor induced by LPS (Bariani et al., 2015). Furthermore, it was also reported that AEA upregulates Cyclooxygenase 2 (COX-2) expression and PGs synthesis in human fetal membranes as well (Mitchell et al., 2008), suggesting an important role of this endocannabinoid in the trigger of parturition.

Our observation on PGs synthesis led us to speculate if molecules involved in myometrial quiescence are also regulated by AEA. Nitric oxide (NO) is responsible for maintaining uterine quiescence (Ledingham et al., 2000) and for regulating placental corticotropin releasing hormone (CRH) release during pregnancy (Roe et al., 1996). This modulating action is important since CRH has been postulated as a molecular clock that determines the length of pregnancy and the time of birth (McLean et al., 1995). Moreover, NO is also one of the main molecules that regulate placental blood flow. In the present work we analyzed NOS activity, and no differences were found between VD and CS samples. In line with these results, it was reported that eNOS expression is similar in both CS and VD placentas (Kakui et al., 2003), and that NOS activity is higher in term CS placentas when compared to the myometrium of the same patient (Al-Hijji et al., 2003). However, it is important to point out that *in vitro* NOS enzymatic activity may not faithfully reflect the *in vivo* NO production because several co-factors participate in its synthesis. Accordingly, NO levels are modulated by several factors, including ECs. We previously demonstrated that AEA contributes to the fine regulation of NO levels in rat placenta (Cella et al., 2008). In the present study we observed that AEA exerts a differential effect on NOS activity. We speculate that during pregnancy, AEA increases NO levels to maintain uterine quiescence and to contribute to a vasodilatation state necessary for adequate oxygen and nutrients supply to the fetus. Conversely, a decrease in NOS activity in VD placentas contributes to releasing the myometrium from relaxants molecules in preparation to receive contractile signals that stimulate the onset of parturition. Besides it would also be critical during labor for preventing excessive bleeding during parturition.

Our results suggest that AEA plays an important role in labor, regulating factors that in turn stimulate either myometrial quiescence or contractility. Furthermore, its action seems to depend on the molecular context of the placenta.

Given that increased AEA levels are considered one of the signals that contribute to the onset of labor, and since the expression of the ES in the placenta during this process remains unknown, we evaluated the expression of members of the ES which are known to be important regulators in this tissue. In agreement with a previous report (Park et al., 2003), we observed that VD placentas express lower FAAH protein levels than CS placentas. In addition, we evidenced a decrease in FAAH enzymatic activity in VD samples, suggesting that placental AEA levels increase with parturition, possibly contributing to the overall increment in AEA tone. The decrease observed in NAPE-PLD mRNA expression in VD samples may be a compensatory mechanism for the presumed rise in placental AEA tone.

Several reports have demonstrated the relevance of CB1 signaling in the regulation of parturition. In fact, defective CB1 signaling in mouse has been associated with preterm onset of labor (Wang et al., 2008). Accordingly, Acone et al. (2009) detected a decrease in CB1 protein levels between non-laboring and laboring placentas. In the present study we did not detect differences in CB1 mRNA and protein levels between samples. The differences with the above-mentioned reports can be attributed to the selection criteria and/or the type of samples included in the study. The distribution of GPCRs on the cell surface and their internalization are regulated by different mechanisms, being the trafficking through Lipid rafts (LRs) a well-described one. A subset of LRs found in cell surface invaginations is called caveolae, in which caveolins (CAVs) are the most relevant structural proteins (Patel and Insel, 2009). Particularly, LRs have a functional effect on CB1 as they regulate not only its partitioning on the membrane and thus the accessibility to the ligand, but also its intracellular trafficking (Sarnataro et al., 2005) via caveolae-mediated endocytosis (Lefkowitz, 1998; Wickert et al., 2018). Due to the importance of CB1 in the regulation of parturition, and since differences in its expression were not observed between placental samples that may explain AEA’s dual effects, we evaluated CB1 immunolocalization. We observed specific CB1 staining mainly in the apical membrane of the STB of CS placentas, while in VD samples CB1 is localized in the STB cytoplasm, although a slight staining was also observed in the basal membrane of the STB. This observation suggests that labor modulates CB1 localization and thus it may impact its response to AEA stimulation.

We therefore analyzed if CB1 is associated to LRs and if it depends on whether the placenta received labor modulating signals. Our DRMs study showed a translocation of CB1 from the apical membrane of the STB to the cytoplasm in CS placentas, and to the basal membrane in VD samples, where CB1 associates to LRs/caveolae as evidenced by its co-localization with FLOTILIN-1 and CAV-1. Our evidence is in line with the fact that LRs can regulate CB1 signaling by controlling its internalization, and of particular interest is the observation by Keren and Sarne (2003) who described that CB1 internalization may be induced by ligand binding. Therefore, we propose that labor modulating molecules, like AEA, may promote CB1 association to LRs/caveolae and thus internalization into the cytoplasm and translocation to the basal membrane of the STB. Whether this is a mechanism for attenuating CB1 signal requires further studies. However, it has been described that CB1 desensitization is a mechanism dependent on LRs/caveolae, in which CB1 is removed from the membrane via endocytosis (discussed in Dainese et al., 2007). In line with this, our observation on the dual effect of AEA on NOS activity could be related to CB1 residing in LRs and thus its lower availability to the ligand. In our study we observed that in non-laboring CS placentas, CB1 mediates AEA effect since incubation with AM251 prevented NOS activity up-regulation, in line with CB1 localizing in the membrane, out of LRs. In contrast, in VD placentas the inhibitory effect of AEA on NOS activity is not mediated by CB1, concomitant with the notion of LRs/caveolae internalizing CB1. It is plausible that VD placentas, exposed to a broad set of factors that regulate labor, may internalize CB1 as a mechanism for attenuating its signaling. The inhibitory effect of AEA on NOS activity could be exerted by a mechanism independent of receptor that involves the participation of LRs, as it was described in other cell types (Biswas et al., 2003; Siegmund et al., 2005; DeMorrow et al., 2007). This could be a possible mechanism of action of AEA in VD placentas.

In summary, we studied the effect of AEA on placentas before and after the onset of labor and we demonstrated that prior to the onset of labor, AEA is relevant for maintaining elevated placental NOS activity and low PGs concentration, contributing to a quiescent state. On the other hand, changes in the expression of placental ES occur with labor. This could result in a decrease of NO production along with an increase in placental PGs concentration, contributing to the contractile signals that stimulate labor. We postulate that CB1 trafficking plays an important role in the labor process, modulating AEA action. Future experiments are required to elucidate the mechanism underlying CB1 trafficking.

Anandamide’s dual action seems to depend on the molecular context of the placenta, in terms of the different scenario stablished by the molecules that regulate labor. We propose that AEA acts as a modulator in the placenta, contributing to the set of signals that participate in the onset of labor at term.

## Data Availability Statement

The raw data supporting the conclusions of this article will be made available by the authors, without undue reservation.

## Ethics Statement

The studies involving human samples were reviewed and approved by the Ethics Committee of the Center for Medical Education and Clinical Research “Norberto Quirno” (No. 684). All patients provided their written informed consent.

## Author Contributions

PA and CA performed experiments and wrote the manuscript. TE analyzed the data. MN and GL provided the placentas and the clinical characteristics of the patients. VH and SM performed DRM’s technique. MF conceived the study. All authors contributed to the article and approved the submitted version.

## Conflict of Interest

The authors declare that the research was conducted in the absence of any commercial or financial relationships that could be construed as a potential conflict of interest.
